# Splenic contraction is enhanced by exercise at simulated high altitude

**DOI:** 10.1007/s00421-021-04637-0

**Published:** 2021-03-08

**Authors:** Angelica Lodin-Sundström, Pontus Holmström, Marcus Ekstam, Daniel Söderberg, Erika Schagatay

**Affiliations:** 1 Department of Health Sciences, Mid Sweden University, Östersund, Sweden; 2 Swedish Winter Sports Research Centre, Östersund, Sweden; 3 Environmental Physiology Group, Department of Nursing Science, Mid Sweden University, Holmgatan 10, 85170 Sundsvall, Sweden

**Keywords:** Spleen size, Oxygen carrying capacity, Normobaric hypoxia, Hemoglobin, Performance

## Abstract

**Purpose:**

Splenic contraction increases circulating hemoglobin (Hb) with advantages during hypoxia. As both hypoxia and exercise have been shown to be important separate triggers of splenic contraction we aimed to investigate if the spleen response to simulated high altitude (HA) is enhanced by superimposing exercise.

**Method:**

Fourteen healthy volunteers (seven females) performed the following protocol in a normobaric environment sitting on an ergometer cycle: 20 min rest in normoxia; 20 min rest while breathing hypoxic gas simulating an altitude of 3500 m; 10 min exercise at an individually set intensity while breathing the hypoxic gas; 20 min rest in hypoxia; and finally 20 min rest in normoxia. Spleen measurements were collected by ultrasonic imaging and venous Hb measured at the end of each intervention.

**Result:**

Mean ± SD baseline spleen volume during normoxic rest was 280 ± 107 mL, the volume was reduced by 22% during rest in hypoxia to 217 ± 92 mL (*p* < 0.001) and by 33% during exercise in hypoxia (189 mL; *p* < 0.001). Hb was 140.7 ± 7.0 g/L during normoxic rest and 141.3 ± 7.4 g/L during hypoxic rest (NS), but increased by 5.3% during hypoxic exercise (148.6 ± 6.3 g/L; *p* < 0.001). Spleen volume and Hb were stepwise changed back to baseline at cessation of exercise and return to normoxia.

**Conclusion:**

Splenic contraction is induced by hypoxia and further enhanced by superimposing exercise, and reduced when exercise ceases, in a step-wise manner, showing that the tonic but partial contraction observed in long-term field expeditions to HA may occur also in the short term. This “graded response” may be beneficial during acclimatization to HA, to cope with moderate chronic hypoxia during rest while allowing additional enhancement of oxygen carrying capacity to overcome short bouts of extreme hypoxia caused by exercise.

## Introduction

Approximately 30–40 million non-acclimatized lowlanders travel to high altitude (HA) each year (Neupane and Swenson 2017). As a result, they are exposed to the decreasing atmospheric pressure and reduced partial pressure of oxygen (PO_2_) with associated reductions in arterial oxygen saturation (SaO_2_) that follow with increasing altitude (Grocott et al. 2009; West 2012), inevitably leading to progressive hypoxia with ascent. In response to the hypoxia acclimatization occurs, which eventually restores O_2_ homeostasis (West 2004). An early elevation in hemoglobin concentration (Hb) occurs, preceding the more long-term effect of acclimatization obtained after weeks by erythropoiesis (West 2012). It has been suggested that this early acclimatization is a result of dehydration associated with increased ventilation at HA, or to changes in plasma volume caused by sodium- and water-regulating hormonal responses to hypoxia (Bärtsch and Saltin 2008; Schmidt 2002). However, studies investigating spleen volume changes with erythrocyte release during hypoxia suggest that splenic contraction could be accountable for at least some of this effect (Schagatay et al. 2005; Richardson et al. 2008; Engan et al. 2014).

In many mammalian species, the spleen functions as an erythrocyte reservoir, recruited during strenuous activities that impose increased demands for O_2_ storage and transport (Barcroft and Stephens 1927; Guntheroth and Mullins 1963; Kramer and Luft 1951; Hurford et al. 1996). Red blood cells from the human splenic reservoir have been found to increase hematocrit (Hct) and Hb during exercise (Sandler et al. 1984; Hoka et al. 1989; Flamm et al. 1990; Laub et al. 1993), eupneic hypoxia (Richardson et al. 2008; Lodin-Sundström and Schagatay 2010), and apneic diving (Hurford et al. 1990; Schagatay et al. 2001; Espersen et al. 2002; Bakovic et al. 2003). The increase in Hb across a series of maximal apneas was associated with prolonged apneic duration in intact participants, whereas splenectomized participants exhibited neither Hb elevation nor prolonged apneic duration (Schagatay et al. 2001).

Splenic contraction occurs also during exercise, resulting in release of stored erythrocytes into systemic circulation (Froelich et al. 1988; Laub et al. 1993). Stewart and associates (2003) found no change in spleen volume following 5, 10, and 15 min of exercise at 60% maximal oxygen uptake ($\dot{\mathrm{V}}{\mathrm{O}}_{2}$_max_), but during maximal exercise, spleen volume was reduced by 56% and concluded that splenic contraction is an active response and may occur in an intensity-dependent manner with heavier exercise. Previous research found that the spleen contracts during 20 min of normobaric hypoxia (F_i_O_2_ 12.8%), leading to an elevation of Hb by 2.1% (Richardson et al. 2008). In another study, comparing splenic contraction in two different hypoxic situations: apnea and 20 min rest during normobaric hypoxia (F_i_O_2_ 14%), authors found that splenic contraction was twice as strong during apnea despite similar SpO_2_ levels (Lodin-Sundström and Schagatay 2010). However, all these studies suggested that the spleen may be important also during HA.

In a recent field expedition to Mount Everest base camp, baseline spleen volume at low altitude was found to be inversely associated with acute mountain sickness symptoms during ascent in non-acclimatized lowlanders (Holmström et al. 2019). This association indicates that individual spleen volume affects tolerance to HA hypoxia, likely by introducing stored erythrocytes into systemic circulation, thereby enhancing O_2_-carrying capacity. Spleen volume and contraction have also been found to be larger in elite climbers summiting Mount Everest compared to recreational trekkers (Schagatay et al. 2020a), but similar to that of successful freedivers who are regularly exposed to intermittent hypoxia (Schagatay et al. 2012). Holmström and colleagues (2020a) also found larger spleen volumes and contraction in Sherpa of highland origin compared to native Nepalese lowlanders, indicating a genetic component involved in spleen size development at HA. However, when the authors in the same study compared differences between Sherpa who live at HA and those who have migrated to low altitude, it was found that the Sherpa living at HA had larger spleen volume, which may indicate that human spleen size also is determined by environmental exposure to HA.

Baseline spleen volume has also been found to decrease in size during incremental ascent at HA (Schagatay et al. 2020b; Holmström et al. 2020b), attesting to a tonic splenic contraction during ascent, with a further volume reduction in response to exercise (Schagatay et al. 2020b) or apnea-induced acute hypoxia (Holmström et al. 2020b). Collectively, these two field studies demonstrated a potentially important role of the splenic contraction at HA. Nevertheless, the combined effects of hypoxia and exercise on splenic contraction need further laboratory investigation to reveal if and to what extent the combined stimuli could induce a progressive, or graded response in the short term, which would seem functional at HA. Splenic contraction can develop fast and is abolished within 10 min (Schagatay et al. 2005), making it a potentially useful mechanism for rapid regulation of the circulating Hb, optimizing the demands on O_2_ transport and those to keep viscosity at an acceptable level to limit cardiovascular stress. The present study explores this possibility by monitoring spleen volume during normoxia and when hypoxia and exercise are superimposed and then removed in a step-wise manner. We hypothesized that (a) rest in normobaric hypoxia would induce splenic contraction and (b) that the superimposed exercise would induce an enhanced splenic contraction resulting in further increase in Hb, and that the response is reversed when these stimuli are subsequently removed.

## Methods

Fourteen healthy volunteers [seven males and seven females: age; 25 ± 3 years; height 177 ± 11 cm; weight 76 ± 14 kg and vital capacity (VC) 5.1 ± 1.1 L] volunteered for the study. The participants were involved in physical training for 6.6 ± 3.5 h per week but none was altitude-acclimatized. Participants were non-smokers but one regularly used snuff tobacco. They received written and oral information about the study and signed an informed consent form. The study complied with the Declaration of Helsinki and was approved by the local human ethics board.

### Procedures and measurements

Each participant performed an individual anaerobic threshold test (IAT) on a cycle ergometer, to establish their individual exercise capacity (load) for the hypoxic test (details below). On a separate occasion after the IAT test, splenic function was measured at rest and during exercise in a simulated hypoxic environment (Hypoxic test). On both occasions participants arrived at the laboratory in a fasted and resting state, after at least 2 h without food or straining physical activity, and at least 1 h without having ingested any beverages.

### Individual anaerobic threshold test

The participants performed a submaximal step-incremental exercise test in normoxia on a cycle ergometer to measure their IAT to establish individual exercise capacity for determination of the load in the hypoxic test. The IAT is defined as the metabolic rate where the elimination of blood lactate (La) during exercise is maximal and equal to the rate of diffusion of La into the blood (Stegmann et al. 1981). Consequently, power outputs above IAT will result in a progressive metabolic acidosis, and exercise time to exhaustion inversely related to the amount of exercise work rate exceeding IAT (Stegmann and Kindermann 1982). The IAT test was based on the maximal step-incremental exercise test by McLellan and Jacobs (1993) involving continuous exercise on a cycle ergometer (Monark ergomedic 828 E, Monark Exercise AB, Vansbro, Sweden). Starting at 30 W (W), the intensity was increased in increments of 30 W every 4 min. Thirty seconds before each incremental step, participants were asked to rate their perceived exertion (RPE) from 6 to 20 (Borg 1998). Heart rate (HR) was measured continuously (Polar RS 800, Polar Electro OY, Kempele, Finland), and capillary blood samples taken from the finger for lactate (La) analysis (Biosen 5140, EKF Diagnostic, Magdeburg, Germany). Participants pedaled with 60 revolutions per minute. The IAT test was terminated when La > 4 mmol/L or when the participant rated 17 on the RPE scale. Even though the IAT, as described by McLellan and Jacobs (1993) and Stegmann and Kindermann (1982) entails maximal effort, these termination endpoints were included as precautionary measures to limit any health risks possibly associated with maximal exercise in hypoxia. That load was used for each individual in the hypoxic test. If the change of La between the final two steps differed by only 1 mmol/L or less, the participants load applied to the hypoxic test was set one step (30 W) below this load.

### Hypoxic exposure and exercise test

Each participant had their anthropometric data and VC measured while standing (Vitalograph Compact 11, Vitalograph, Buckingham, England), with the larger of 3 VC measurements used. An intravenous catheter (BD Venflon Pro, Becton Dickinson Infusion Therapy AB, Helsingborg, Sweden) was placed in the antecubital region to collect serial blood samples.

**Fig. 1:**
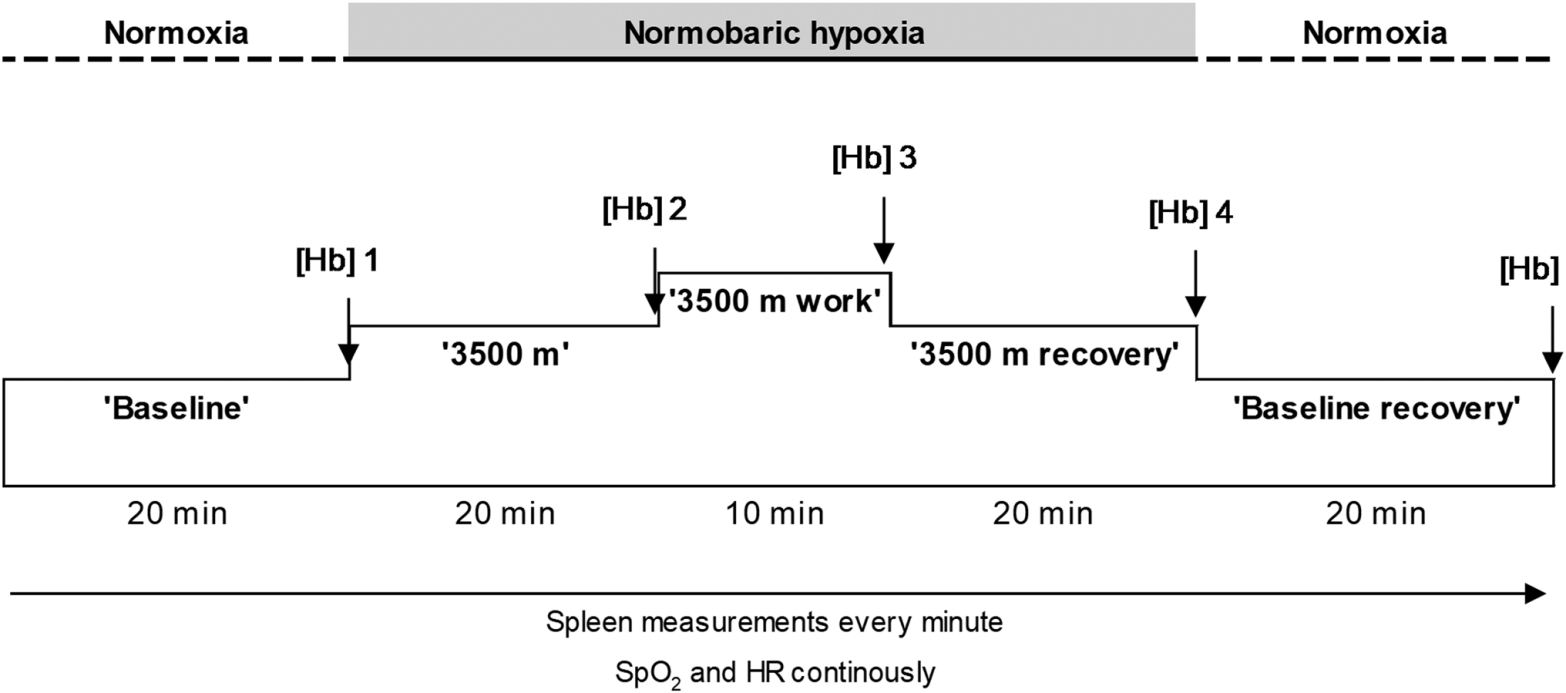
Experimental procedure

Participants rested for 20 min sitting on a cycle ergometer to achieve a homeostatic state in normoxia (‘Baseline’). Hypoxia was induced by placing a non-rebreathing mask over the participants’ nose and mouth allowing continuous breathing of 14% oxygen in nitrogen for 20 min (Hypoxico, Hypoxico Inc., New York USA) simulating an altitude of 3500 m (‘3500 m’). Thereafter, participants performed 10 min of cycling with the constant load previously determined in the IAT test at a frequency of 60 rev*min^−1^ while continuously breathing hypoxic air through the mask (‘3500-work’). The exercise was followed by 20 min rest on the cycle ergometer, with participants still breathing hypoxic air through the mask (‘3500-recovery’). Finally the mask was removed and the participants continued to rest in normoxia for another 20 min in the same position (‘Baseline-recovery’; Fig. 1).

#### Measurements

Spleen measurements were collected every minute throughout the experiment using ultrasonic imaging (Mindray DP-6600, Shenzhen Mindray Bio-Medical Electronics Co., Ltd., Shenzhen, China; Fig. 1). Peripheral oxygen saturation (SpO_2_) and HR were continuously recorded using a pulsoxymeter (Biox 3700e, Ohmeda, Madison, WI, USA) with a probe on the index finger. The continuously registered parameters were stored via a multichannel data acquisition system (BioPac Systems Inc., Goleta, CA, USA).

Venous blood samples were collected for Hb measurements during the last minute of each of the five exposures (Fig. 1). The volume of blood removed per participant was less than 25 mL including waste, with approximately 10 mL of sterile isotonic NaCl solution infused for rinsing the catheter. In one participant capillary blood samples were collected due to blood clot in the catheter.

### Data analysis

The triaxial measurements of splenic length (*L*), width (*W*), and thickness (*T*) were used to calculate volume with the Pilström equation [*Lπ*(*WT* − *T*^2^)/3] (Schagatay et al. 2005). Values from the final 2 min of each intervention were compared to baseline and among interventions.

Venous blood samples were analyzed in triplicate using an automated unit (Micros 60 Analyzer, ABX Diagnostics, Montpellier, France) and in cases where a deviation of triplicate samples of > 3 g/L occurred, a fourth was analyzed. In one participant all blood samples were analyzed using a portable Hb analyzer (Hemocue Hb 201+, Hemocue AB, Ängelholm, Sweden) due to capillary sampling.

### Statistical analysis

Data are reported as mean ± standard deviation (SD), unless otherwise stated. IBM SPSS statistics (version 25 for windows) was used to run appropriate statistical analysis. Normality distribution of data was tested using Kolmogorov–Smirnov and Shapiro–Wilk tests (*p* > 0.05). Assessments of interaction effects between the independent variables [treatment (‘baseline; 3500 m’, ‘3500-work’; ‘3500-recovery’; ‘Baseline-recovery’) and sex] on the dependent variables (spleen volume, Hb, SpO_2_, and HR) was conducted by a 2 × 5 two-way mixed ANOVA with Bonferroni corrections for repeated measures. Assessments of associations between dependent variables were conducted with bivariate correlation tests using Pearson product moment correlation coefficient (*r*). Meaningfulness of treatment effects was estimated by the standardized mean difference [Cohen’s *d*, effect size (ES)] computed as the mean difference divided by the pooled SD. ES was presented along with 95% confidence intervals ([CI]). An ES of 0.0–0.3 was considered a small effect, 0.4–0.7 medium and > 0.8 a large effect (Lee 2016). Statistically significant differences were assumed at *p* < 0.05.

## Results

All participants successfully completed the experiment protocol. The two-way mixed ANOVA revealed a significant treatment effect on all dependent variables (spleen volume, Hb, SpO_2_, and HR). Nevertheless, there was no two-way interaction effect between males and females on the dependent variables during the experimental intervention (NS), indicating that these variables change equally between sexes in response to hypoxia and exercise. Therefore, all data for responses are presented with sexes pooled. Females had significantly smaller spleen volumes throughout the experiment (*p* = 0.009) with a baseline volume of 218 ± 73 mL compared to males at 342 ± 103 mL (*p* = 0.024; ES = 1.39 [0.14–2.44]). There was no difference in Hb, HR or SpO_2_ between females and males throughout the experiment (NS).

### Spleen response

**Fig. 2:**
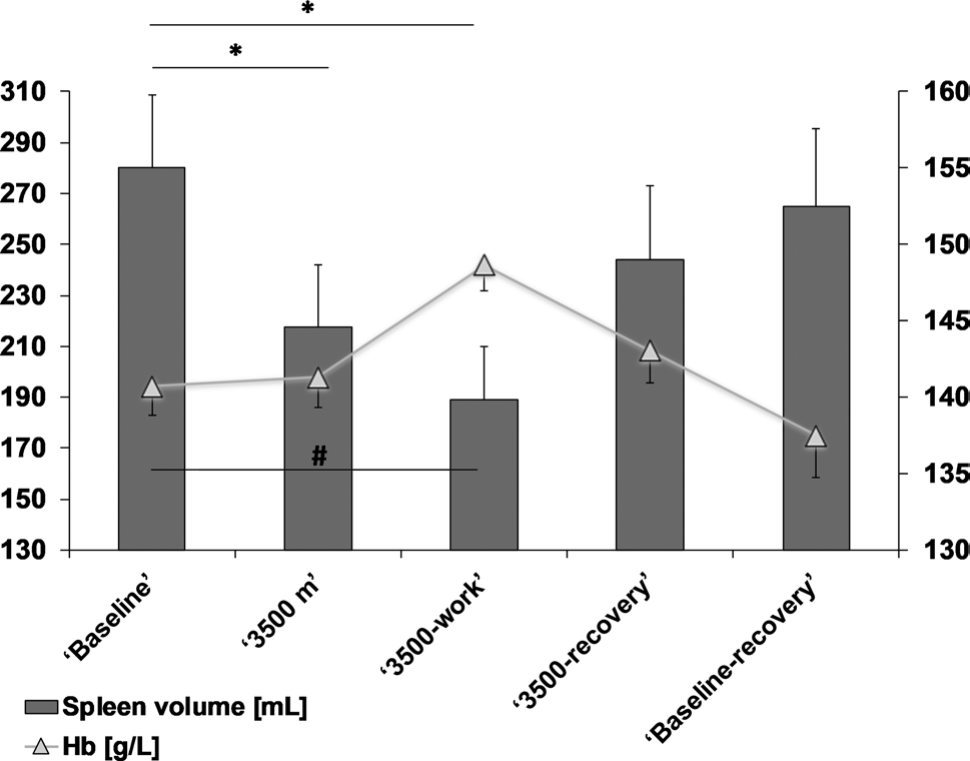
Spleen volume and Hb changes during the experimental procedure. Standard error of the mean (SEM) is used to indicate the sample distribution. Significant difference at *p* < 0.001 is indicated by * for spleen volume and # for Hb

Baseline spleen volume in normoxic air was 280 ± 107 mL. Splenic contraction developed gradually during ‘3500 m’ and had decreased to 217 ± 92 mL (by 22%) after 20 min (Fig. 2; *p* < 0.001; ES = 0.63 [− 0.15 to 1.37]). The contraction was enhanced after ‘3500-work’, wherein spleen volume was reduced to 189 ± 78 mL (by 33%) after 10 min of exercise (Fig. 2; *p* < 0.001; ES = 0.97 [0.16–1.72]). Spleen volume increased during ‘3500-recovery’ to 244 ± 108 mL (NS; ES = 0.33 [− 0.42 to 1.07] at 20 min. Spleen volume was further increased to 265 ± 113 mL in normoxia during ‘baseline-recovery’, where it reached baseline values within 14 min (Fig. 2; NS; ES = 0.14 [− 0.61 to 0.87]).

Baseline spleen volume was strongly associated with the magnitude of the contraction throughout the experiment: ‘3500 m’ (*r* = 0.942, *p* < 0.001); ‘3500-work’ (*r* = 0.887, *p* < 0.001); ‘3500-recovery’ (*r* = 0.932, *p* < 0.001); ‘Baseline-recovery’ (*r* = 0.902, *p* < 0.001).

### Hematological response

Baseline Hb was 140.7 ± 7.0 g/L at the end of ‘Baseline’ normoxic rest, and after 20 min at ‘3500 m’ Hb was at 141.3 ± 7.4 g/L (Fig. 2; NS; ES = 0.00 [− 0.74 to 0.74]). At the end of ‘3500-work’, Hb had increased to 148.6 ± 6.3 g/L, (by 5.3%) from ‘Baseline’ (Fig. 2; *p* < 0.001; ES = 1.23 [0.39–2.00]). By the end of ‘3500-recovery’, Hb was 143.0 ± 7.8 g/L (NS; ES = 0.27 [0.49–1.00]) and it was restored by the end of ‘Baseline-recovery’ at 137.5 ± 10 (NS; ES = 0.35 [− 0.41 to 1.08]).

### Cardiovascular parameters

Baseline SpO_2_ and HR were 97 ± 1% and 78 ± 9 bpm, respectively. After 20 min at ‘3500 m’, SpO_2_ had decreased to 88 ± 4% (by 9%; *p* < 0.001; ES = 3.09 [1.92–4.08]) and HR had increased to 82 ± 10 bpm (by 5%; NS; ES = 0.42 [0.34–1.16]). By the end of exercise SpO_2_ had decreased to 77 ± 6% (by 13% from ‘3500 m’; *p* < 0.001; ES = 2.16 [1.17–3.02]) and HR increased to 125 ± 29 bpm (by 52% from ‘3500 m’; *p* < 0.001; ES = 1.98 [1.03–2.82]). By the end of the 20 min recovery at sea-level ‘Baseline-recovery’, all parameters had returned to pre-exposure baseline values (SpO_2_: 97 ± 1%; HR: 79 ± 11 bpm; NS).

## Discussion

We investigated the combined effect of hypoxia and exercise on splenic contraction during simulated HA equivalent to 3500 m (F_i_O_2_ 14%) in non-acclimatized lowlanders. The principal findings were that during rest, normobaric hypoxia induced a partial splenic contraction, which was enhanced with superimposed exercise and subsequently reduced again with return to rest in hypoxia, and further reduced by return to normoxic air. The spleen volume decreases during hypoxia and exercise was accompanied by Hb elevation, and the return to baseline spleen size with a reduction in Hb. Thus, the spleen responded in a step-wise manner to the progressively increased and decreased stimuli, which  demonstrates that the spleen has the ability to fine tune the amount of circulating red cells in the short term to optimize between metabolic needs and cardiovascular load (sheer stress). This is the first laboratory study confirming earlier long-term field observations suggesting that, when non-acclimatized lowlanders are exposed to HA, a partial tonic splenic contraction is induced on which additional stimuli may induce yet a more powerful response (Richardson and Schagatay 2007; Holmström et al. 2020b; Schagatay et al. 2020b). The present study shows such a fine-tuned graded response may also be induced in the short term.

Richardson and Schagatay (2007) found an attenuated apnea-induced Hb increase during ascent to 5100 m, whereby they concluded that hypobaric hypoxia at HA causes a tonic splenic contraction that most likely leads to elimination of further apnea-induced Hb increases. Holmström and colleagues (2020b) measured spleen volume and Hb during incremental ascent at HA in non-acclimatized lowlanders. They reported that baseline spleen volume decreased by approximately 14% per 1000 m ascent, which was also negatively associated with baseline Hb, further attesting to a tonic splenic contraction during ascent at HA. However, in addition to supporting the existence of a tonic splenic contraction in non-acclimatized lowlanders during HA exposure, with a putative effect to enhance oxygen uptake, our findings contributes new knowledge of a short-term splenic ability to transiently fine-tune circulating erythrocytes in an acute phase, likely to optimize between increased demands on O_2_ transportation to muscles and hypoxia sensitive organs during bouts of exercise and reducing viscosity during rest to reduce cardiovascular stress. While the level of hypoxia is similar, it should be noted that normobaric hypoxia is different from hypobaric hypoxia, which may result in differences in hypoxia-induced responses of some variables e.g., ventilation, NO, and fluid retention (Coppel et al. 2015).

Our data displayed a significant splenic contraction at rest in hypoxia, which is in line with previous research that suggests that hypoxia is an important trigger of the splenic contraction (Richardson et al. 2008; Lodin-Sundström and Schagatay 2010). The magnitude of 22% reduction in spleen volume observed in the present investigation during rest at simulated altitude of 3500 m, was in the range of responses previously reported, i.e., a decrease in spleen volume by 18% after resting participants were exposed to normobaric hypoxia corresponding to an altitude of 4100 m, which also resulted in a Hb elevation of 2.1% (Richardson et al. 2008). Lodin-Sundström and Schagatay (2010) reported a splenic contraction by 16% during rest in normobaric hypoxia, corresponding to an altitude of 3300 m. Since resting spleen volume and contraction vary between individuals (Prassopoulos et al. 1997), the different reported magnitudes of spleen contraction may be due to individual differences between subjects in the relatively small groups studied.

While splenic contraction occurs during exercise (Laub et al. 1993; Stewart et al. 2003), apnea (Schagatay et al. 2005), normobaric hypoxia (Richardson et al. 2008; Lodin-Sundström and Schagatay 2010) hypobaric hypoxia (Holmström et al. 2020b; Schagatay et al. 2020b) and during handgrip exercise (Frances et al. 2008), the mechanisms behind human splenic contraction have not been fully clarified. Nevertheless, hypoxia appears to be a major factor inducing the response (Richardson et al. 2008; Lodin-Sundström and Schagatay 2010). The splenic nerve is almost entirely (98%) composed by sympathetic nerve fibers and may be affected by increased hypoxia-induced sympathetic output. More rapid changes in spleen volume have also been reported in apneic situations, with immediate onset of apnea before hypoxia has developed, which has been addressed to withdrawal of inhibitory baroreceptor activity and reduced cardiac output, resulting in a significant fall in mean arterial pressure increasing the sympathetic output (Bakovic et al. 2003; Palada et al. 2007). Thus, both rapid neural and progressive chemoreceptor input may elicit spleen contraction, with the latter being a likely candidate in this study. The severe exercise-induced O_2_ desaturation indicates systemic hypoxemia, conceivably the chemoreceptors may regulate the response, where PO_2_ below a certain level may likely cause a more pronounced splenic contraction if the hypoxemic stress is severe enough. With rest in mild hypoxia the effects are minor, but with onset of exercise an additional neural input is likely involved.

The enhanced splenic contraction with exercise reflects the ability of the spleen to continuously regulate circulating Hb to the varying demands on the O_2_ transportation system associated with different levels of exertion and consequent muscle O_2_ demands. This fine-tuned regulation may allow sustained performance at HA even though the physiological demands increase with exercise. Dane et al. (2006) observed that the spleen blood serves an important function for O_2_ transportation during exercise in mammals with high-aerobic capacity like dogs, and showed that the elevated Hb due to splenic contraction contributed to an increased diffusion of O_2_ from the lung to the blood and from blood to tissue. This suggests that the spleen is an important reservoir also during exercise in situations when the body is already under stress, and shows that the spleen was only partially contracted during 3500 m altitude simulation.

Maximum aerobic performance is reduced in hypoxia (Bärtsch and Saltin 2008), hence exercise in hypoxia entails working at a higher relative workload compared to normoxia. Therefore, even light work will impose a substantial stress to the system. It is, therefore, difficult to distinguish between the effect of enhanced hypoxia in itself and those imposed by the workload. It has previously been suggested that splenic contraction develops in an intensity-dependent manner during exercise (Stewart et al. 2003). This is the first study to demonstrate this intensity-dependent contraction as a graded response to hypoxia as well.

Regardless of input, the reversible nature of the response shows that the temporary enhancement of circulating Hb is not likely due to hemoconcentration as a result of extravasation of plasma, which is in accord with the previous studies (Schagatay et al. 2001; Bakovic et al. 2003). Hb concentrations may increase during exercise, suggested to be primarily due to rapid plasma water reductions, resulting from a transient fluid shift from intravascular to extravascular compartments (Jacobs et al. 2012). Nonetheless, elevated levels of erythrocytes by splenic contraction have also previously been suggested to contribute to the Hb concentration increase during exercise at sea level (Jacobs et al. 2012; Laub et al. 1993; Stewart et al. 2003), resulting in a 4–6% elevation in Hct (Laub et al. 1993; Stewart et al. 2003) and O_2_-carrying capacity improvements by approximately 10% (Schmidt and Prommer 2010). This is consistent with our findings of a reversible Hb increase during exercise in hypoxia by 5.3% and we, therefore, suggest that the splenic contraction that occurs during exercise in normobaric hypoxia contributes substantially to the Hb increase.

### Methodological considerations

One limitation is that the IAT test was performed in normoxia for the test conducted in hypoxia. However, since all subjects served as their own control, this is not expected to have influenced the conclusions. The current study observed a marginal not significant Hb elevation when entering hypoxia compared to normoxia at rest. This was unexpected as previous research has displayed a 2.1% Hb elevation resulting from splenic contraction in normobaric hypoxia equivalent to 4100 m (F_i_O_2_ 12.8%) ascribed to hypoxia-induced splenic contraction (Richardson et al. 2008) but as our simulated altitude of 3500 m was lower, this could have resulted in a too small drop in SpO_2_. In addition, in a previous study, baseline spleen contraction and Hb elevation during rest was small and the main effect was seen with exercise (Schagatay et al 2020a, b). Another possible cause of the minor Hb effect, despite a significant spleen contraction is that we used the same time point for spleen measurements and blood samples, but there could be a circulatory delay of the Hb effect. The small sample size also did not allow a reliable conclusion to be done from the correlation analysis. The fact that previously reported effects of normobaric hypoxia at rest on Hb were also small and individual differences in Hb are large means that changes may possibly be undetected in a small sample, although we found significant stepwise splenic volume changes.

## Conclusions

We found that the splenic contraction induced by hypoxia during rest is enhanced when exercise is superimposed, with a concomitant increase in circulating Hb. The enhanced response with exercise is likely a result of the further decrease in SpO_2_. With normoxia both responses are fully recovered to baseline within 20 min. This step-wise response suggests that the spleen may provide a means for a rapid and fine-tuned control of circulating erythrocytes with exercise at altitude, and a rapid reversal of this effect at exercise termination, limiting the period with increased viscosity. This clearly confirms results from earlier studies done in the field at varying environmental conditions, and shows these changes also occur in the short term. Together with earlier field studies this report supports the view that the moderate tonic splenic contraction with hypoxia at rest may contribute to coping with HA before any effects on Hb of erythropoiesis has developed, likely resulting in an increased hypoxia tolerance at HA. The remaining capacity for splenic contraction is recruited only during the transient excessive stress imposed by e.g., exercise.

## Abbreviations

ES: Effect size
HA: High altitude
Hb: Hemoglobin concentration
HR: Heart rate
IAT: Individual anaerobic threshold
La: Blood lactate
O_2_: Oxygen
PO_2_: Partial pressure of oxygen
RPE: Rating of perceived exertion
SaO_2_: Arterial oxygen saturation
SpO_2_: Peripheral oxygen saturation
VC: Vital capacity
VO_2max_: Maximal oxygen uptake
